# Overdiagnosis in organised mammography screening in Denmark. A comparative study

**DOI:** 10.1186/1472-6874-9-36

**Published:** 2009-12-22

**Authors:** Karsten J Jørgensen, Per-Henrik Zahl, Peter C Gøtzsche

**Affiliations:** 1 The Nordic Cochrane Centre, Rigshospitalet, Dept 3343, Blegdamsvej 9, DK-2100 Copenhagen, Denmark; 2 Norwegian Institute of Public Health, PO Box 4404, Nydalen, N-0405, Oslo, Norway

## Abstract

**Background:**

Overdiagnosis in cancer screening is the detection of cancer lesions that would otherwise not have been detected. It is arguably the most important harm. We quantified overdiagnosis in the Danish mammography screening programme, which is uniquely suited for this purpose, as only 20% of the Danish population has been offered organised mammography screening over a long time-period.

**Methods:**

We collected incidence rates of carcinoma in situ and invasive breast cancer in areas with and without screening over 13 years with screening (1991-2003), and 20 years before its introduction (1971-1990). We explored the incidence increase comparing unadjusted incidence rates and used Poisson regression analysis to compensate for the background incidence trend, variation in age distribution and geographical variation in incidence.

**Results:**

For the screened age group, 50 to 69 years, we found an overdiagnosis of 35% when we compared unadjusted incidence rates for the screened and non-screened areas, but after compensating for a small decline in incidence in older, previously screened women. Our adjusted Poisson regression analysis indicated a relative risk of 1.40 (95% CI: 1.35-1.45) for the whole screening period, and a potential compensatory drop in older women of 0.90 (95% CI: 0.88-0.96), yielding an overdiagnosis of 33%, which we consider the most reliable estimate. The drop in previously screened women was only present in one of the two screened regions and was small in absolute numbers.

**Discussion:**

One in four breast cancers diagnosed in the screened age group in the Danish screening programme is overdiagnosed. Our estimate for Denmark is lower than that for comparable countries, likely because of lower uptake, lower recall rates and lower detection rates of carcinoma in situ.

## Background

Overdiagnosis in cancer screening is defined as the detection of cancers that would otherwise not have been detected in the remaining life-span of the individuals [1]. It is mainly caused by the detection of slow-growing cancers that do not manifest clinically before people die from other causes [2], but may also be due to identification of borderline malignancies, or cancers that were bound to regress [3,4].

Overdiagnosis is arguably the most important harm of screening, as healthy people are being diagnosed with and treated for cancer unnecessarily, which carries great personal costs, both physically and psychologically [2].

Overdiagnosis is an unavoidable consequence of mammography screening [2]. It is also well documented for other cancers, e.g. lung cancer, neuroblastoma and prostate cancer [5]. Although about 60% of men in their 60's have cancer lesions in their prostate in autopsy studies, the observed incidence is much smaller and the lifetime risk of dying from prostate cancer is only 3% [5]. Some of these otherwise undetected cancers will become diagnosed if screening is implemented, and this is an important reason that screening for prostate cancer is discouraged in many Western countries [5]. Overdiagnosis is also a problem with mammography screening, but it has been been omitted in most information material intended to help women make informed decisions about participation [6-8].

Overdiagnosis in mammography screening has been documented in systematic reviews of the randomised trials. Screening led to a 31% increase in the use of breast cancer surgery, which included a 20% increase in the use of mastectomies [9]. We have recently quantified the extent of overdiagnosis in breast cancer screening programmes in Manitoba, New South Wales, Norway, Sweden and the UK and found 52% overdiagnosis, including carcinoma in situ lesions (CIS) [10]. There were large and sustained increases in breast cancer incidence when screening was introduced, with only small or absent compensatory decreases among older, previously screened women. A compensatory decrease in incidence is required, if the incidence increase in the screened group is due only to advancement of the time of diagnosis (lead-time) [11]. Data on CIS lesions are often lacking in articles that describe indicence trends [10], although CIS is mainly diagnosed through screening and contributes substantially to overdiagnosis, as these lesions currently constitute 20% of breast cancers detected at screening in the UK and more in the USA [12,13], and although less than half of them progress to invasive breast cancer [2]. A limitation of our systematic review was that there were no concomitant control groups without screening and we therefore used linear projections of pre-screening rates to estimate the overdiagnosis [10].

Estimating overdiagnosis can be particularly difficult in countries that do not have an organised screening programme and therefore have no well-defined pre- and post-screening period, e.g. the USA. Also, in the USA, there is no control group without screening, as all areas have screening in private practices. Comparisons with younger and older age groups is also unreliable in this case, as women are recommended to be screened from they are very young, and as there is no upper age limit. Contrary to this, the Danish breast cancer screening programme provides a unique opportunity for estimating overdiagnosis, as there has been a period of 17 years (1991-2007) where only about 20% of potentially eligible women have been invited to screening with mammography, in two separate administrative regions, with a well-defined target age group and starting point. Partly because of an intensive debate about the balance between the benefits and harms of screening, the Danish administrative regions have prioritised their resources differently. A national screening policy has now been adopted and is currently being implemented.

We compared breast cancer incidence in areas with and without organised screening. We studied whether the increase in incidence in women who were offered screening was compensated by a drop in breast cancer incidence when the women passed the age limit for screening, and compared with the development in women in the same age group in areas without screening, and with that in younger women. We corrected the estimates of overdiagnosis for differences in age distribution, geographical differences in pre-screening incidence rates and changes in background incidence with Poisson regression analyses.

## Methods

We retrieved data on breast cancer incidence in females during 1971-2003 from the Danish Cancer Registry at the National Board of Health (data were not yet available for 2004-2007). The data we received were aggregated by geographical region and into 5-year age groups. Data on population size for each year, region and age group were obtained from Statistics Denmark [14]. We included carcinoma in situ, as these lesions are treated surgically as if they were invasive cancers.

Organised mammography screening of women aged 50-69 years began April 1st 1991 in Copenhagen municipality, November 1st 1993 in Funen County, and June 1st 1994 in Frederiksberg municipality. The Frederiksberg programme, which comprised comparatively few women, was incorporated into the Copenhagen programme January 1st 1997 [15]. In 2003, there were 115,270 women aged 50-69 years old in the screened areas (54,933 in Copenhagen and Frederiksberg and 60,337 in Funen), and 551,778 in areas without organised screening [14].

The attendance rates were 63% in Copenhagen and 83% in Funen in the fourth screening round [16], which are lower than in Sweden, Norway, Finland and the UK [2]. The recall rates per incidence round were 4.3% in Copenhagen and 1.3% in Funen [15], which are also lower than in comparable countries.

The population in Copenhagen and Funen is comparable with the rest of Denmark regarding age distribution and socioeconomic status. Copenhagen is the largest city, but the second largest is in the non-screened areas, and there are rural areas in Funen County, like in the rest of Denmark. The Danish population is one of the most homogenous in the world.

We first explored the incidence increase using the observed rates, without adjustments. We calculated the average incidence rate ratio between screened and non-screened areas for the screened age group. We then subtracted the compensatory decline in older, previously screened women (ages 70-79 years) to estimate overdiagnosis, after calculating the absolute number of diagnoses that the incidence rates corresponded to. Two years after screening was introduced, women aged 70 and 71 would have been offered screening once, as women are called for screening every second year. After four years, women aged 72 and 73 would have been offered screening once, and women aged 70 and 71 would have been offered screening twice. After 10 years, all women would have been offered screened previously (which is in 2001 in Copenhagen and in 2003 in Funen), with the younger ages having been offered screening more than once. We would therefore expect to see a trend towards declining incidence in the age group 70-79 years, beginning early on after the onset of screening. We also used simple linear regression to estimate incidence trends before and after the introduction of screening [10].

We used Poisson regression analyses to obtain more reliable estimates of overdiagnosis, with confidence intervals, and adjusted for differences in age distribution using 5-year age groups, and for the fact that Funen introduced screening 3 years after Copenhagen, and for geographical differences in incidence, using the pre-screening period as reference.

We also used Poisson regression analysis to quantify the compensatory drop in incidence in women ages 70-79 years. The Poisson regression model was adjusted only for age and included a trend parameter, starting in 1998, because 7 in 10 women in Copenhagen in this age group, and 4 in 10 women in Funen, would then have been offered screening previously. We used the incidence in the rest of Denmark as reference to compensate for the increasing background incidence in this age group.

The statistical analyses were performed using Egret version 2.0.3 and graphs were made in Microsoft Excel 2000.

## Results

Data were available from 13 years of organised mammography screening (1991-2003). In women aged 50-69 years, 5,189 cases of breast cancer (of which 6% were CIS) were diagnosed during 1,342,836 woman-years in areas offering screening (average 386/100,000 woman-years), and 17,686 cases (3% CIS) were diagnosed during 6,191,609 woman-years in areas not offering screening (average 286/100,000 woman-years) (Table 1).

**Table 1:** Number of breast cancers, number of women, and incidence rates in screened and non-screened areas, before and after screening started, and during the last three years of observation.

		Screened areas			Non-screened areas		
		
		1971-1990 No screening	1991-2003 Screening	2001-3 Screening	1971-1990	1991-2003	2001-3
**Breast cancers**	35-49 years	2,110	1,684 (4% CIS)	381 (3% CIS)	8,668	7,228 (5% CIS)	1,741 (4% CIS)
	
	50-69 years	5,846	5,189 (6% CIS)	1,282 (6% CIS)	16,263	17,686 (3% CIS)	4,922 (2% CIS)
	
	70-79 years	3,258	2,058(3% CIS)	383 (2% CIS)	7,256	6,483 (2% CIS)	1,767 (2% CIS)

**Person years**	35-49 years	1,759,614	1,317,024	314,322	7,827,731	6,038,527	1,412,069
	
	50-69 years	2,737,925	1,342,836	326,946	8,223,810	6,191,609	1,565,967
	
	70-79 years	1,195,296	606,034	117,077	2,743,410	2,100,884	481,608

**Per 100,000**	35-49 years	120	128	121	111	120	123
	
	50-69 years	214	386	392	198	286	314
	
	70-79 years	273	340	327	264	309	367

**Figure 1:**
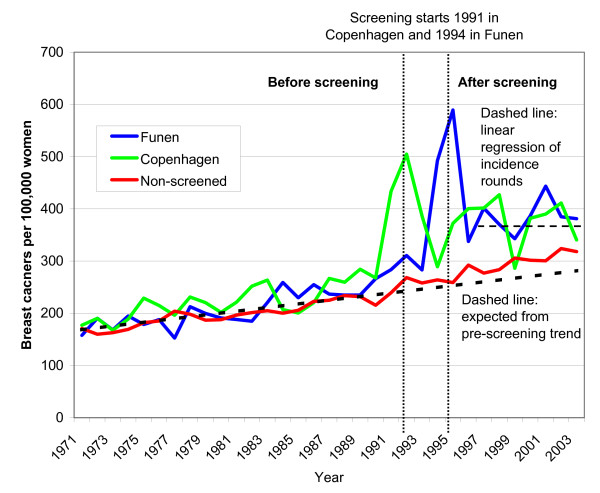
**Unadjusted incidence of in situ and invasive breast cancers per 100,000 women ages 50-69 years in areas without mammography screening and in Copenhagen and in Funen**.

Among women in the screened age group, pre-screening breast cancer incidence increased at a stable rate from 1971-1990 (Fig. 1), with slightly higher rates in the screened areas than in the non-screened areas (average 214 vs. 198 breast cancers per 100,000 person-years) (Table 1). There were also slightly higher rates in the screened areas in the age groups 35-49 years and 70-79 years in this period (Table 1).

The breast cancer incidence doubled in Copenhagen in 1991 and in Funen in 1994, when screening was introduced (Fig. 1). After the first round of screening, the breast cancer incidence in screened areas was about 30% higher than in the non-screened areas, and more when compared with the expected incidence projected from the pre-screening incidence. Using a linear regression analysis for the screened areas for the incidence rounds 1996-2003, there were 36% more breast cancers than expected in 2003 in the screened areas (Fig. 1). In the non-screened areas, the breast cancer incidence was also somewhat higher than expected (Fig. 1).

**Figure 2:**
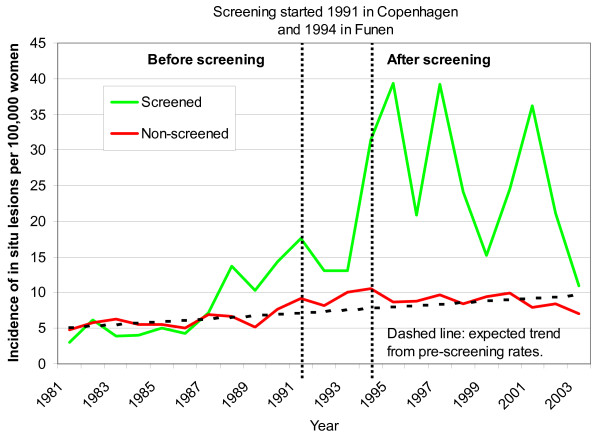
**Unadjusted incidence of *in situ *lesions only, per 100,000 women ages 50-69 years, in areas without mammography screening and in Copenhagen and in Funen**.

The screening activity in the age group 50-69 years is reflected in the detection rates of CIS (Fig. 2). There were increases of several hundred per cent in the screened areas, both compared to the rates in the non-screened areas, and to expected rates (Fig. 2). Rates of CIS increased somewhat in the screened areas before the introduction of organised screening, beginning in 1988 (Fig. 2), likely because of opportunistic screening.

**Figure 3:**
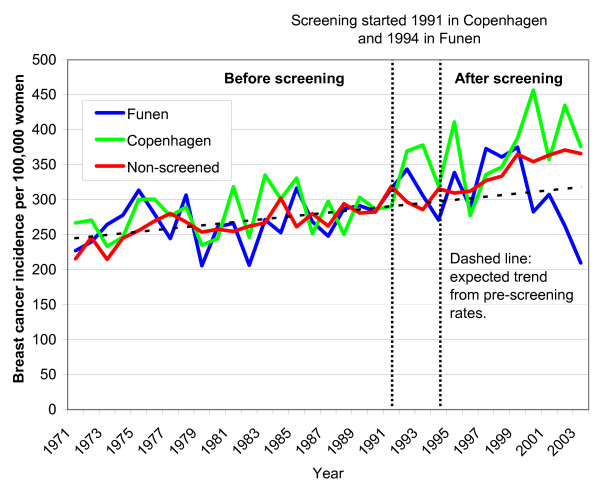
**Unadjusted incidence of *in situ *lesions and invasive breast cancers per 100,000 women ages 70-79 years in areas without mammography screening and in Copenhagen and in Funen**.

In the age group 70-79 years, breast cancer incidence also increased steadily from 1971-1990 (Fig. 3). Breast cancer incidence in Copenhagen increased more than expected after the introduction of screening in 1991 (Fig. 3). In Funen, breast cancer incidence also increased after the introduction of screening, but there was a drop towards the end of the observation period (Fig. 3). Breast cancer incidence in the non-screened areas also increased more than expected after 1991 (Fig. 3).

**Figure 4:**
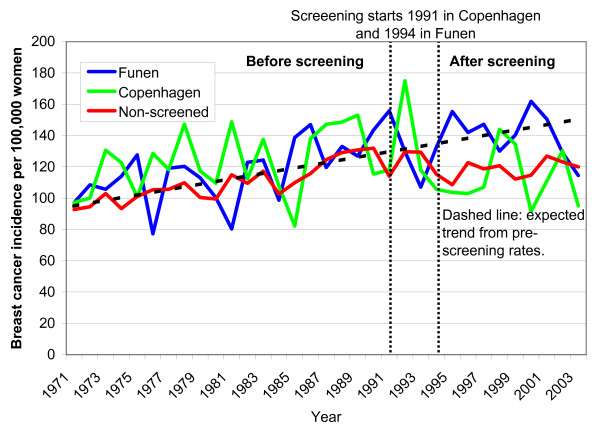
**Unadjusted incidence of in situ and invasive breast cancers per 100,000 women ages 35-49 years in areas without mammography screening and in Copenhagen and in Funen**.

In women aged 35-49 years, breast cancer incidence increased steadily in the period 1971-1990. It then stabilized in Copenhagen and the non-screened areas, but not in Funen (Fig. 4).

In the 13 years of screening, there were 1,343 extra breast cancers detected in the screened areas compared with the non-screened areas in women aged 50-69 years, when comparing the unadjusted incidence rates (386 vs. 286 breast cancers per 100,000 women in 1,342,836 woman years, Table 1), and there were 182 *extra *cancers detected in the screened areas collectively, compared with the non-screened areas, among women aged 70-79 years, i.e. no compensatory drop (Table 1). The 1,343 extra cancers are equivalent to 35% overdiagnosis in the screened population (Table 2). If we compare only the years 2001-3, where there was a drop in incidence in Funen among women aged 70-79 years, there were 255 extra cancers detected in the age group 50-69 years, 47 of which may have been compensated. This conservative analysis means that there were 208 uncompensated extra cancers in the screened population, or 19% overdiagnosis (Tables 1 and 2). However, the data set is small and random fluctuation might therefore explain that this estimate is lower than that for the main analysis.

**Table 2:** Number of extra cancers in screened women (ages 50-69 years), number of extra cancers compensated in previously screened women (ages 70-79 years), and unadjusted estimates of overdiagnosis in Denmark.


	**1991-2003**	**2001-3**

**Extra breast cancers**	1,343	255

**Cancers compensated in women aged 70-79 years**	None (182 surplus cancers)	47

**Overdiagnosed breast cancers**	1,343	208

**Overdiagnosis**	35%	19%

### Poisson regression analyses

In our Poisson regression analysis, where we took potential biases into account, we found a risk ratio of 2.07 (95% CI: 1.94-2.21) for the first rounds, and 1.34 (95% CI: 1.29-1.40) in the following period (Table 3). The weighted average was 1.40 (95% CI: 1.35-1.45).

**Table 3:** Poisson regression analysis with estimated risk ratios for in situ and invasive breast cancer combined in Denmark adjusted for increasing background incidence, geographical differences in pre-screening incidence, and delayed start of screening in Funen.

	RR (95% CI)
1971 (reference)	1.0 (APC 0,37%, 0.30-0.45)
Screening effects, women aged 50-69 years	
First round	2.07 (1.94 to 2.21)
Later rounds	1.34 (1.29 to 1.40)
Women aged 70-74 years	
1998-2003	0.89 (0.80 to 0.98)
Women aged 70-79 years	
1998-2003	0.90 (0.85-0.96)
Pre-screening difference (screened vs. rest)	
All ages	0.90 (0.89 to 0.92)

We found a risk ratio of 0.90 (95% CI: 0.85-0.96) in women aged 70-79 years in Copenhagen and Funen combined in the period 1998-2003, using Poisson regression analysis. Using the same model for the age group 70-74 years, where a compensatory drop would appear first and be most pronounced, we found a risk ratio of 0.89 (95% CI: 0.80-0.96) (Table 3). We used Poisson regression to test for a trend towards a linearly accellerating drop in incidence in women 70-79 years, as such a trend would increase the likelihood that screening caused the decline. Again, we chose 1998 as our starting point and repeated the analysis for the age group 70-74 years to increase the chance of detection. We did not find such a trend, neither for the age group 70-79 years (P = 0.50), nor for the age group 70-74 years (P = 1.00), the annual percentage change was 0.37% (95% CI: 0.30-0.45).

Forty percent more breast cancers in the screened population and a 10% compensatory drop in previously screened women means that there were 5, 189 - (5,189/1.40) = 1,483 extra breast cancers detected in the period 1991-2003, and that 2,058 × 0.1 = 206 of these were later compensated (Table 1), which gives 33% overdiagnosis.

## Discussion

When we adjusted for regional differences in incidence and age distribution, and compensated for a decline in incidence in older, previously screened women, we found 33% overdiagnosis in Denmark. This is lower than the 52% we estimated for other countries in our systematic review of organised screening programmes, which did not include Denmark [10]. The likely reasons for this are that the Danish screening programme has low recall rates, e.g. only 1.3% per round in Funen [15], low detection rates for CIS (6% of diagnoses, Table 1), because of a deliberately conservative attitude towards detection of microcalcifications [17], and comparatively low uptake, e.g. 63% in Copenhagen [16].

We did not take opportunistic screening in the non-screened areas into account in our Poisson regression analyses, and we might therefore have underestimated overdiagnosis. We abstained from this adjustment, as the level of opportunistic screening in the non-screened areas is difficult to estimate. Increasing rates of CIS indicate that there was opportunistic screening in Copenhagen and Funen from about 1988, but that there was little opportunistic screening in the rest of Denmark (Fig. 2). Another study confirmed our findings and estimated that opportunistic screening outside the organised programme covered only 10% of the women [18], and noted that it was difficult to differentiate between diagnostic and screening mammograms using available statistics. CIS has been reported to be poorly registered outside the screening programmes [17] and increased little (Figure 2), whereas the incidence of breast cancer overall increased more than expected in the non-screened areas from 1991 (Fig. 1). This increase is partly due to overdiagnosis caused by opportunistic screening, but could also partly reflect an increase in background incidence, although the stable incidence rates in the age group 35-49 years speak against this (Figure 4). As we compared screened and non-screened regions, general changes in the background incidence would not affect our estimate of overdiagnosis, which is a strength of our study, compared to using projections of pre-screening incidence rates in the screened regions only [10,17].

In 2003, at the end of our observation period, practically all women aged 70-79 years in Copenhagen and Funen had been offered screening several times at an earlier age and had therefore contributed to the observed incidence increase in the age group 50-69 years earlier on. In other words, the cohort of women aged 70-79 years in 2003 consisted entirely of women who were 60-69 years earlier on in our observation period and who had therefore been offered screening previously. In the absence of overdiagnosis, the incidence in the age group 70-79 years should therefore drop more and more with increasing follow-up. However, there was no drop in incidence rates in Copenhagen and it is contrary to screening theory that we failed to detect a trend towards progressively lower incidence rates in previously screened women [11]. There was a drop in Funen after about 7 years with screening (Fig. 3), but as there were fewer breast cancers in Funen, and as the incidence increased more than expected in this age group in Copenhagen, the combined drop is negligible. A compensatory drop should have occurred earlier in Copenhagen than in Funen, which speaks against that the drop in Funen is related to screening. We consider it unlikely that longer follow-up would change these findings, as we were also unable to demonstrate important compensatory declines in countries with longer follow up than Denmark [10]. The data material is small enough that the drop in Funen could be a random fluctuation and more follow up is required to establish this. Until this is available, we therefore consider our conserative estimate of 19% overdiagnosis based on only the last observation years as less reliable.

In the screened areas, breast cancer incidence in the age group 50-69 years was higher than in the age group 70-79 years throughout the period with organised screening (compare Fig. 1 and 3). Mammography screening has dramatically changed the shape of the age-specific breast cancer incidence curve: it was increasing with age prior to screening, but now has a maximum in the age group 50-69 years. Furthermore, because there are many more women in the age group 50-69 years than in the age group 70-79 years (ratio 2.3 to 1, see Table 1), the drop in the incidence rate in the age group 70-79 must be much larger than the increase in the incidence rate in the age group 50-69 years, if all the extra breast cancers detected through screening are to be compensated. Even if no breast cancers were diagnosed in the age group 70-74 years in 2003, it would not account for the extra breast cancers detected in the screened age group. This is not compatible with common expectations of an average lead-time of 2-3 years [10,19,20] and indicates that a large part of the observed incidence increase must be due to other causes.

There were minor differences between the screened and non-screened regions in pre-screening incidence (Table 1 and 3). Such differences were expected, as large cities comprised a greater proportion of the screened areas, and as breast cancer incidence is generally higher in cities. There was also a higher incidence in the non-screened areas than expected from the linear projection of the pre-screening trend (Figure 2). This is partly due to opportunistic screening, as discussed above, but could also be due to other factors that increase the breast cancer risk, such as HRT (hormone replacement therapy). However, while it is likely that some women in the non-screened areas would seek opportunistic screening once it was available to others, there is no good reason why the increase should occur simultaneously with the introduction of screening if it were due to HRT. The findings of our recent systematic review on overdiagnosis also speak strongly against a general, non-screening related rise in the background incidence, over the pre-screening trend [10]. In the countries we studied, the abrupt increase in incidence always occurred simultaneously with screening, despite the fact that screening was introduced a decade apart in the various countries [10]. Further, the increase was by far most predominant in the invited age range, despite the fact that this also varied between countries [10].

We did not compare closed cohorts. Influx of patients to regions with screening could boost incidence in the screened age groups and in older age groups, leading to overestimation of overdiagnosis. However, the mobility between Danish regions in the age group 50-79 years is limited, and our findings are in good agreement with those from comparable countries such as Sweden and the UK, which has nation-wide screening and can be considered more similar to a closed cohort [19], and where we found an overdiagnosis of about 50% [10]. It could be argued that in the beginning of the observation period following the introduction of screening, we compare a screened cohort with a women who have never been offered screening. However, this is not so much a limitation of our cohorts as a choice that allows us to document if there was a trend towards a decline in the incidence rate of breast cancer, as the proportion of women in the age group 70-79 years that had previously belonged to the screened age cohort of women (women aged 50-69 years) increased. There was no such trend, and at the end of our observation period, we compared practically only currently screened women with previously screened women. Using only the last observation year would be unreliable, as the data would be prone to random fluctuations given our comparatively small sample size.

The absence or the small magnitude of a compensatory drop in previously screened women we found here, and in our previous review [10], questions the central premise of breast cancer screening. It shows that very little of the surplus of cancers observed in the screened age group can be due to advancement of the time of diagnosis (lead-time) for lethal cancers where screening might be beneficial. Previous estimates of average lead-times of 2-3 years [19,20] must therefore be wrong.

It has been suggested by comparison of left- with right-sided irradiation that radiotherapy may double not only the mortality from heart disease, but also that from lung cancer [21], although technological improvements may have diminished these harms to some extent. Lung cancer is currently rivalling breast cancer as the leading cancer related cause of death among women in the Western world and heart disease is the major cause of death in women. It is therefore of interest that an effect of screening on all-cause mortality has not been demonstrated. The randomised trials were not powered to detect a difference in all-cause mortality, but as more than half a million women participated in the trials, this at least indicates a limited absolute benefit of the intervention [9].

More surprisingly, there is no indication that screening lowers all-cancer mortality, including breast cancer mortality. The relative risk was 1.00 (95% CI 0.96-1.05) in the randomised trials [9], although with the commonly stated 30% reduction in breast cancer mortality with screening, the expected relative risk for all-cancer mortality would be 0.95, which is below the confidence interval of what was actually found [9].

Svendsen et al. have previously concluded that the Danish data do not provide evidence of overdiagnosis of invasive breast cancer, or that it was of limited magnitude [17]. The authors reported that the observed incidence rate in the screening period in Copenhagen and Funen, considered separately from each other, was within the 95% confidence interval of the expected rate, which they projected from the pre-screening rates using regression analysis. Our linear regression analysis of the pre-screening rates in the two screening regions combined indicated that a total of 3,901 women with breast cancer would be expected, whereas we observed 5,189 cases during the 1,342,836 woman-years from 1991-2003. This yields an incidence rate ratio of 1.33, or 33% more breast cancers than expected, with a 95% confidence interval of 1.28-1.39. However, Svendsen et al. not only separated calculations of their confidence intervals for Copenhagen and Funen, they also calculated the confidence interval for each year of observation individually. Further, they left out diagnoses of CIS from their calculations. Thus, by splitting the data, Svendsen et al. only found a non-significant difference, and this formed the basis for their conclusion. We believe the study by Svendsen et al. does not provide evidence against overdiagnosis, and other authors have excluded it from their review on overdiagnosis [22].

Some of the same authors have previously claimed that, after the prevalence peak in the first rounds, the breast cancer incidence in a screened population should decline rapidly to the level expected without screening, if there were no overdiagnosis and a closed cohort was studied [23]. The Danish authors claimed that the incidence returned to the pre-screening level and that organised mammography screening could therefore operate without overdiagnosis [23]. However, they did not present a statistical analysis in support of this and have later published data for Copenhagen that are compatible with elevated incidence levels in the screened age group following the introduction of mammography screening [24].

In another study [20], some of the same authors discussed complexities in the estimation of overdiagnosis and used a lead-time model for calculating overdiagnosis in Sweden. This is an unreliable approach because current estimates of lead-time disregard overdiagnosis and its substantial influence on such calculations. Adjusting for lead-time using these estimates will therefore underestimate overdiagnosis. Most importantly, lead-time models assume that a very pronounced drop in incidence rates occurs in previously screened age groups, e.g. a drop of about 50% has been suggested, based on an assumed lead-time of 5 years [11]. However, such large drops have never occurred [10], as we have also shown here for Denmark.

## Conclusions

The 33% overdiagnosis we found means that one in four breast cancers diagnosed in a screened population is overdiagnosed. This is lower than the 52% we have previously estimated for other mammography screening programmes, likely because the Danish programme has low uptake, a deliberately conservative attitude towards microcalcifications, and low recall rates. Despite these precautions, the level of overdiagnosis is still disturbingly high and it leads to overtreatment and great physical and psychological harms for those who experience it. It is therefore important that women receive balanced information that makes is possible for them to decide on a rational basis whether screening is right for them. Unfortunately, the official information leaflets women receive when they are invited to screening do not tell them about overdiagnosis and overtreatment [8]. We have therefore published an evidence-based leaflet [25] that has been translated into several languages, and which conveys the message that it is not clear whether breast screening does more good than harm [9].

## Competing interests

The authors declare that they have no competing interests.

## Authors' contributions

KJJ and PCG conceived the project. KJJ collected data and wrote the first draft. PHZ performed statistical analyses. PHZ and PCG revised the manuscript.

## Pre-publication history

The pre-publication history for this paper can be accessed here:

http://www.biomedcentral.com/1472-6874/9/36/prepub
